# Supplementary material for: The Effect of Domain-Specific Sitting Time and Exercise Habits on Metabolic Syndrome in Japanese Workers: A Cross-Sectional Study
Source: Int J Environ Res Public Health. 2020 May 30;17(11):3883. doi: 10.3390/ijerph17113883 (PMC7312666; doi:10.3390/ijerph17113883)
Supplement: Supplementary file 1 [file ijerph-17-03883-s001.pdf]

Table S1. Analysis of logistic regression analyses examining the risk of MetS for each domain-specific ST and habitual exercise. (n = 5,530)

	Total ST			Occupational ST			Leisure-time ST on workday			Leisure-time ST on holiday			Habitual exercise	
	short (≦ 8.9h)	middle (9.0h - 11.9h)	long (12.0h ≦)	short (≦ 5.2h)	middle (5.3h - 8.1h)	long (8.2h ≦)	short (≦ 2.6h)	middle (2.7h - 4.1h)	long (4.2h ≦)	short (≦ 8.0h)	middle (8.1h - 11.5h)	long (11.6h ≦)	Yes (n =1,474)	No (n =4,056)
MetS														
Model 1	1.00	1.17 (0.91-1.51)	**1.38 (1.09-1.76)**	1.00	1.04 (0.82-1.33)	1.00 (0.79-1.28)	1.00	0.98 (0.77-1.25)	1.15 (0.90-1.46)	1.00	1.23 (0.96-1.59)	**1.56 (1.22-1.98)**	1.00	**1.35 (1.08-1.68)**
Model 2	1.00	1.09 (0.84-1.40)	1.24 (0.97-1.59)	1.00	0.96 (0.75-1.22)	0.87 (0.68-1.12)	1.00	0.99 (0.78-1.27)	1.23 (0.96-1.57)	1.00	1.23 (0.95-1.58)	**1.43 (1.12-1.83)**	1.00	**1.44 (1.15-1.80)**

Values are presented as odds ratio (OR) and (95% confidence interval). The significant OR (95% confidence interval) are indicated in the table by boldface values. Model 1 was not adjusted; Model 2 was adjusted for age, sex, smoking (0: ex-smoker and non-smoker, 1: smoker), alcohol (0: non-consumption, 1: once or twice per week, 3 –5 times per week, and ≥6 times per week) and shift work (0: absence of shift work, 1: presence of shift work).

Table S2. Analysis of logistic regression analyses examining the risk of MetS for combined habitual exercise and tertiles of each domain-specific ST. (n = 5,530)

	Habitual exercise			No-habitual exercise		
Total ST	Short (n =635)<br>≦ 8.9h	Middle (n = 657)<br>9.0h - 11.9h	Long (n = 512)<br>12.0h ≦	Short (n = 1,207)<br>≦ 8.9h	Middle (n = 1,189)<br>9.0h - 11.9h	Long (n = 1,330)<br>12.0h ≦
Model 1	1.00	1.16 (0.74-1.81)	1.08 (0.67-1.75)	1.19 (0.80-1.77)	1.41 (0.96-2.08)	**1.75 (1.20-2.54)**
Model 2	1.00	1.05 (0.67-1.64)	0.95 (0.58-1.54)	1.25 (0.84-1.86)	1.39 (0.94-2.05)	**1.64 (1.12-2.39)**
Occupational ST	Short (n =556)<br>≦ 5.2h	Middle (n = 645)<br>5.3h - 8.1h	Long (n = 603)<br>8.2h ≦	Short (n = 1,288)<br>≦ 5.2h	Middle (n = 1,205)<br>5.3h - 8.1h	Long (n = 1,233)<br>8.2h ≦
Model 1	1.00	0.98 (0.62-1.54)	0.92 (0.57-1.47)	1.25 (0.84-1.84)	1.35 (0.92-2.00)	1.31 (0.89-1.93)
Model 2	1.00	0.83 (0.52-1.31)	0.75 (0.47-1.22)	1.27 (0.85-1.87)	1.27 (0.86-1.88)	1.15 (0.77-1.70)
Leisure-time ST on workday	Short (n =599)<br>≦ 5.2h	Middle (n = 645)<br>5.3h - 8.1h	Long (n = 603)<br>8.2h ≦	Short (n = 1,288)<br>≦ 5.2h	Middle (n = 1,205)<br>5.3h - 8.1h	Long (n = 1,233)<br>8.2h ≦
Model 1	1.00	0.82 (0.52-1.29)	1.01 (0.64-1.60)	1.13 (0.77-1.65)	1.33 (0.92-1.94)	1.33 (0.92-1.92)
Model 2	1.00	0.69 (0.43-1.09)	0.88 (0.55-1.40)	1.17 (0.80-1.71)	1.23 (0.84-1.80)	1.22 (0.84-1.78)
Leisure-time ST on holiday	Short (n =798)<br>≦ 8.0h	Middle (n = 562)<br>8.1h - 11.5h	Long (n = 444)<br>11.6h ≦	Short (n =1,117)<br>≦ 8.0h	Middle (n = 1,221)<br>8.1h - 11.5h	Long (n = 1,388)<br>11.6h ≦
Model 1	1.00	1.22 (0.79-1.89)	1.12 (0.69-1.80)	1.13 (0.78-1.65)	1.38 (0.96-1.98)	**1.86 (1.33-2.62)**
Model 2	1.00	1.23 (0.79-1.91)	1.05 (0.65-1.70)	1.26 (0.86-1.85)	**1.49 (1.03-2.14)**	**1.84 (1.30-2.59)**

Values are presented as odds ratio (OR) and (95% confidence interval). The significant OR (95% confidence interval) are indicated in the table by boldface values. Model 1 was not adjusted; Model 2 was adjusted for age, sex, smoking (0: ex-smoker and non-smoker, 1: smoker), alcohol (0: non-consumption, 1: once or twice per week, 3 –5 times per week, and ≥6 times per week) and shift work (0: absence of shift work, 1: presence of shift work).
